# Anterior Commissural White Matter Fiber Abnormalities in First-Episode Psychosis: A Tractography Study

**DOI:** 10.1016/j.schres.2015.01.037

**Published:** 2015-02-07

**Authors:** Zora Kikinis, Jennifer Fitzsimmons, Chandler Dunn, Mai-Anh Vu, Nikos Makris, Sylvain Bouix, Jill M. Goldstein, Raquelle I. Mesholam-Gately, Tracey Petryshen, Elisabetta C. del Re, Joanne Wojcik, Larry J. Seidman, Marek Kubicki

**Affiliations:** a Department of Psychiatry, Brigham and Women's Hospital, Harvard Medical School, Boston, MA, USA; b Psychiatry and Neurology Departments, Massachusetts General Hospital, Harvard Medical School, Charlestown, MA, USA; c Departments of Psychiatry and Medicine, Brigham and Women's Hospital, Harvard Medical School, Boston, USA; d Beth Israel Deaconess Medical Center, Massachusetts Mental Health Center, Public Psychiatry Division, Harvard Medical School, Boston, MA,USA; e Psychiatric and Neurodevelopmental Genetics Unit, Department of Psychiatry and Center for Human Genetic Research, Massachusetts General Hospital, Boston, MA, USA; f Stanley Center for Psychiatric Research, Broad Institute of MIT and Harvard, Cambridge, MA, USA; g VA Boston Healthcare System, Brockton, MA, USA; h Department of Psychiatry, Massachusetts General Hospital, Harvard Medical School, Boston, MA, USA

**Keywords:** first-episode schizophrenia, anterior commissure (AC), Diffusion Tensor Magnetic Resonance Imaging (dMRI), fractional anisotropy (FA), radial diffusivity (RD), tractography

## Abstract

**Background:**

The Anterior Commissure (AC) is an important interhemispheric pathway that connects contralateral temporal lobes and orbitofrontal areas. The role of the AC is not yet well understood, although abnormalities in this white matter tract have been reported in patients diagnosed with chronic schizophrenia. However, it is not known whether changes in the AC are present at earlier stages of the disease.

**Methods:**

Diffusion Magnetic Resonance Images (dMRI) were acquired from 17 First Episode Schizophrenia Patients (FESZ) and 20 healthy controls. The AC was reconstructed using a streamline tractography approach. DMRI measures, including Fractional Anisotropy (FA), Trace, Axial Diffusivity (AD) and Radial Diffusivity (RD) were computed in order to assess microstructural changes in the AC.

**Results:**

FA was reduced, while trace and RD showed increases in FESZ. AD did not show differences between groups.

**Conclusion:**

The observed changes in these dMRI measures, namely reductions in FA and increases in trace and RD, without changes in AD, likely point to myelin abnormalities of this white matter tract, and provide evidence of white matter pathology extant in the early phases of schizophrenia.

## 1. Introduction

It has been suggested that schizophrenia is associated with a functional disconnectivity of the brain (Fornito et al., 2011; Friston and Frith, 1995; Mesulam and Geschwind, 1978; Wernicke, 1906). According to this theory, functionally specialized brain regions are highly interconnected through white matter tracts and a disconnection in these tracts may lead to dysfunction in the brain. Connectivity of the brain can now be studied in vivo by using functional imaging in resting-state brain networks (Karbasforoushan and Woodward, 2012), and an abnormal interhemispheric connectivity has been reported in schizophrenia (Guo et al., 2013). The commissural fibers are important for the transfer of information, including complex cognitive information between the hemispheres (Moldrich et al., 2010; van der Knaap and van der Ham, 2011). Additionally, the coordinated transfer of information is essential for appropriate cerebral functions. The cerebral hemispheres are connected by the corpus callosum (CC) and the anterior commissure (AC), as well as by the posterior and the hippocampal commissures. The largest of these pathways is the CC, which transfers the majority of interhemispheric information. However, when the CC becomes dysfunctional, the AC plays a compensatory role by providing comprehensive inter-hemispheric communication. This has been demonstrated in several studies, first, in an experiment on rhesus monkey with lesions in the CC (O'Reilly et al., 2013), second, in individuals with agenesis of the CC (Risse et al., 1978), and, third, in individuals with epilepsy and complete resection of the CC (Spencer, 1988). These studies consistently show that the functionality of motor, language and cognition remained unaffected as long as the AC was intact. Interestingly, **reduced** interhemispheric functional connectivity and structural abnormalities in the CC have been reported in chronic and in FESZ patients (Guo et al., 2013; Henze et al., 2012; Knochel et al., 2012; Kubicki et al., 2005; Samartzis et al., 2014; Whitford et al., 2010), suggesting a pivotal role of the AC in the etiology of this disorder.

The AC and the CC each interconnect specific cortical areas. The major brain areas connected by the AC are the temporal pole, inferior temporal gyrus, superior temporal gyrus, parahippocampal gyrus, fusiform gyrus, amygdala, orbitofrontal cortex and olfactory bulb (Barbas and Pandya, 1984; Cipolloni and Pandya, 1985; Demeter et al., 1990; Di Virgilio et al., 1999; Schmahmann, 2006; Turner et al., 1979). The AC is a relatively small fiber tract (diameter of 3.5-4.3 mm^2^) (Allen and Gorski, 1992) that is visible on structural MRI and on diffusion tensor magnetic resonance images (dMRI) in the midsagittal plane. In comparison to the CC, AC makes up only about 1% of the CC area (Foxman et al., 1986). Due to the small size, until now, the AC of the human and primate brains has mostly been explored in post-mortem preparations, and only very recently using dMRI *in vivo*. Using the dMRI technique the AC was reconstructed in healthy subjects, in adolescents born preterm and in adolescents with bipolar disorder (Catani et al., 2002; Northam et al., 2012; Patel et al., 2010; Saxena et al., 2012). Interestingly, one of the dMRI studies showed that AC fibers can be tracked all the way into the occipital and parietal lobes in humans, suggesting a wider distribution of the AC axons in humans than in primates (Patel et al., 2010). To the best of our knowledge, only one dMRI study, conducted in our laboratory, has explored the AC in patients diagnosed with chronic schizophrenia (Choi et al., 2011) but not in earlier stages of the disease.

DMRI is a well established, noninvasive method to explore brain white matter integrity, and has been applied to thousands of studies, of which about 200 have focused on schizophrenia. It is sensitive to changes in extracellular water movement, and measures used most frequently in clinical DTI studies include Fractional Anisotropy (FA), Trace, Axial Diffusivity (AD) and Radial Diffusivity (RD). FA is a measure of the directionality of water diffusion (Basser et al., 1994) and has highest values in white matter regions of highly myelinated and organized fibers (Beaulieu, 2002). The two other dMRI measures, AD and RD, are believed to be related more, but still indirectly, to neurobiological properties of white matter within the fiber bundle. AD is a measure of diffusion along the length of the axon (e.g., the largest eigenvalue), whereas RD is the average of the two measures of diffusion orthogonal to the axon. Trace is the sum of the AD and the RD and describes the overall diffusivity of water molecules. Trace is increased in the case of any tissue damage, but also as a result of vasogenic edema and inflammation. A series of animal experiments has shown that demyelination of axons is reflected by increased RD and decreased FA, with AD unchanged (Song et al., 2003; Song et al., 2005). Subsequently, dMRI measures have been applied to evaluate the micropathology of multiple diseases.

DMRI has proven useful in investigating changes in white matter prior to and throughout the disease course of schizophrenia (Kubicki and Shenton, 2014). When compared to a healthy control group, changes in dMRI measures describe biological changes in brain tissues by the mean of altered water diffusion. Prior to disease onset, decreases in FA and AD (with no changes to RD and trace) were reported in individuals with 22q11.2 deletions syndrome (22q11DS), a syndrome with an incidence of 30% for developing schizophrenia (Kikinis et al., 2012). These changes in dMRI measures have been interpreted as indicative of changes in axonal integrity, likely a consequence of neurodevelopmental abnormalities. At the time immediately before (clinical high risk phase) and immediately after the first episode of schizophrenia (FESZ), increases in trace and decreases in FA have been interpreted as a possible sign of tissue edema, suggestive of neuroinflammation (Clemm von Hohenberg et al., 2014; Pasternak et al., 2012). It is further hypothesized that neuroinflammation occurs temporarily and gives way later to myelin degeneration in the chronic phase of schizophrenia. Indeed, decreases in FA and associated increases in RD, the signature of abnormalities of the myelin sheath, have repeatedly been reported in FESZ and in patients with chronic schizophrenia (e.g. (Ashtari et al., 2007; Seal et al., 2008; Whitford et al., 2010))

Of interest to the hypothesis of disconnectivity are the structural abnormalities observed in interhemispheric pathways in schizophrenia. Structural abnormalities in the CC have been reported in chronic and in FESZ patients (Henze et al., 2012; Knochel et al., 2012; Kubicki et al., 2005; Samartzis et al., 2014; Whitford et al., 2010), and is one of the most consistent findings in schizophrenia (e.g.(Melonakos et al., 2011)). Since lesion studies suggest that dysfunctional CC is not sufficient to result in cognitive deficits, as long as AC is preserved (O'Reilly et al., 2013; Risse et al., 1978; Spencer, 1988), we explored whether patients with schizophrenia evince abnormalities in AC at the time of first episode. Thus in this study we used dMRI and tractography to reconstruct the AC tract in FESZ patients and healthy controls. We compared dMRI measures, including FA, trace, AD and RD, between the two groups, and assessed microstructural abnormalities of the AC tract in FESZ.

## 2. Methods

### 2.1 Subjects

Seventeen first episode schizophrenia patients (13 male/4 females) were diagnosed with FESZ and twenty (15 males/5 females) control subjects were recruited as part of the larger Boston Center for Intervention Development and Applied Research (CIDAR) study (www.bostoncidar.org). Diagnoses were based on a diagnostic interview using the Structured Clinical Interview for the DSM-IV-TR, Research Version (SCID) for ages 18 years and older, (First, 2002), or the Kiddie-SCID for subjects 13-17 years of age (Hien D, 1994). The patients were either not medicated (two patients) or medicated with antipsychotic medication (fifteen patients). All medication dosages were converted to chlorpromazine equivalents (Stoll, 2009; Woods, 2003). The IQ was estimated using the Wide Range Achievement Test 4 (WRAT-4) (Wilkinson and Robertson, 2006), which among others measures the subject's ability to read and comprehend sentences. The WRAT-4 Reading sub-score assesses the subject's IQ at young age before the onset of schizophrenia. The severity of schizophrenia symptoms was assessed in patients using the Positive and Negative Syndrome Scale (Andreasen, 1983, 1984). Further demographic and clinical information for patients and controls is given in Table 1.This study was approved by the local IRB committees at all participating institutions.

### 2.2 Image acquisition and processing

Diffusion weighted images (DWI) were acquired on a 3 Tesla System GE Echospeed (General Electric Medical Systems, Milwaukee, WI) scanner. The scan parameters were: TR 17,000 ms, TE 78 ms, FOV 24 cm, 144×144 matrix and 1.7 mm slice thickness. Acquisitions used 51 gradient direction with b=900s/mm and 8 baseline scans with b=0. The whole brain was imaged in 85 axial slices. The raw data was processed for noise reduction, eddy current and head motion. Tensors were estimated by the least squares method (Basser and Jones, 2002) using 3DSlicer software version 3.6.2 version (http://www.slicer.org/) (Pieper S., 2004; Pieper S., 2006).

### 2.3 Tractography of the AC

**Fig 1:**
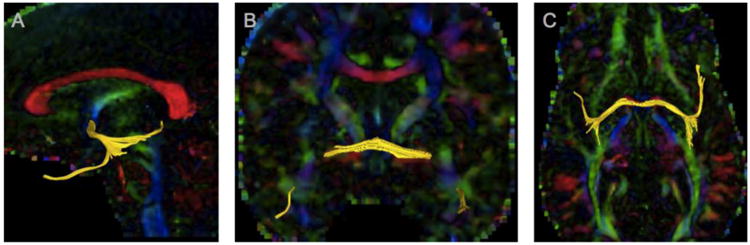
A,B,C. Reconstruction of the anterior commissure fiber tract from dMRI Anterior Commissure (AC) is represented in yellow; with color by orientation map in the background. View of the midsagittal slice (A), anterior view of the AC tract interconnecting both hemispheres as well emerging through the temporal stem and reaching towards the temporal pole (B), inferior view (C).

The AC was reconstructed using ROIs (Region-Of-Interest) based streamline tractography using 3Dslicer software (Fig. 1). Individual ROIs were drawn as described by Choi (Choi et al., 2011), and further modified by adding an exclusion ROI to remove stray fibers. In summary, we manually drew two inclusion and one exclusion ROI on color-by-orientation label maps over the AC. The location of the ROIs and AC was based on the following anatomy: the AC crosses the hemispheres in the mid sagittal plane from left to right and appears as red voxels on color-by-orientation label maps just below the ascending part of fornix. The first inclusion ROI was placed in the axial view over the AC where it appears in red due to its extension across the hemispheres. In order to capture the AC of both cerebral hemispheres, the ROI was drawn on all slices where the AC was visible, which usually varied between one and three slices. However, since fornix and parts of the internal capsule travel through this area, many extraneous fibers were captured when tractography was seeded from the first ROI. To exclude these extraneous fibers, a second inclusion ROI was placed in the midsagittal plane and was drawn over the existing ROI in such a way that it extended the border of the first ROI by two voxels. By adding a second inclusion ROI we limited streamlines to only those that interconnect both hemispheres and are specific to AC. Commissural fibers, other than AC, were further excluded by an exclusion ROI drawn in the mid-sagittal slice over the entire brain, except for where the second inclusion ROI was placed. Voxels defined by the first inclusion ROIs were used as seeds to perform streamline tractography. The resulting fiber bundle was then restricted by the second inclusion ROI and the exclusion ROI. Streamline tractography followed the direction of the major eigenvector, based on the Runge-Kutta protocol (Basser and Jones, 2002) and was set to a spacing between seeds of 1 mm, and with all other settings as defaults (minimum linear measure to start at 0.3, minimum length of the fiber of 10 mm, maximal length of the fiber of 800 mm, stopping mode linear measure of 0.1, stopping curvature if the radius of curvature becomes smaller than 0.8 mm and an integration step of 0.5 mm). Finally, tracts were visualized and checked for consistency. This approach resulted in a precise and accurate reconstruction of the part of the AC where it crosses the hemispheres. The final output of the tractography analysis was the mean value of FA, trace, AD and RD over the bundle. Reliability was performed by two raters (CD, ZK), both blind to diagnosis. Inter-rater reliability was calculated for 8 random cases and the intraclass correlation coefficient (ICC) was 0.98.

### 2.4 Statistical Analysis

We used the Statistical Package for Social Sciences (PASW, version 17.0; SPSS Inc., Chicago, IL, USA) for data analysis to calculate group differences for socio-demographic data and for dMRI measures of the AC. We used Effect Size Calculator (http://www.uccs.edu/∼faculty/lbecker/) to estimate the effect size. Effect size was determined based on the t and df from independent *t*-test.

## 3. Results

### 3.1 Demographics

Patients and control subjects were group-matched on age, gender, education, parental socioeconomic status (PSES), handedness and WRAT-4 Reading test, the latter an estimate of basic reading skills and intelligence prior to disease onset (Table 1). None of the demographic characteristics correlated with the dMRI measures (FA, trace or RD) in either the patient or the control groups, suggesting that dMRI measures were independent of these measures.

### 3.2 Reconstruction of the AC tract

Using dMRI data and streamline tractography we successfully identified the AC (Fig. 1) in all 17 patients, and in 16 out of 20 control subjects (AC not reconstructed in 3 males, 1 female). Consistent with previously published research, the AC reconstructed in this study projects mostly to the temporal pole, but also in a much smaller percentage of fibers to temporal, parietal and occipital lobes. This distribution of streamlines was explored visually and checked for neuroanatomical consistency based on previously published postmortem and dMRI findings (Barbas and Pandya, 1984; Di Virgilio et al., 1999; Patel et al., 2010; Schmahmann, 2006).

### 3.3 Abnormalities in the AC in FESZ group

**Fig 2:**
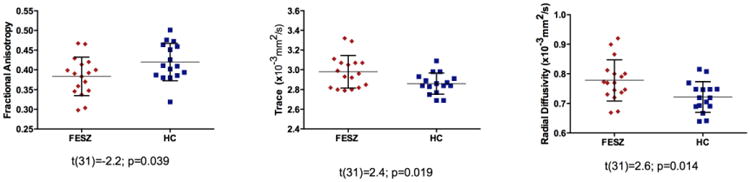
Group differences between patient and control groups Anterior commissure was reconstructed from dMRI using the tractography approach and dMRI measures, such as fractional anisotropy (FA), trace and radial diffusivity (RD) extracted. Statistically significant group differences between first episode schizophrenia (FESZ) patients and healthy controls (HC) are presented. T and p values from independent t-test are reported.

DMRI measures were averaged over the entire AC tract and the mean FA, mean trace, mean AD and mean RD determined. These dMRI values were compared between the FESZ and the healthy control (HC) groups using independent samples t-tests. There was a statistically significant group difference in the values for FA, trace and RD (Fig. 2). No significant group differences were found for AD. In the patient group, the mean of the FA values were decreased (mean FA value 0.42+/-0.05 for HC, 0.38+/-0.05 for FESZ), trace values were increased (mean trace 2.9 +/-0.1 ×10^-3^ mm^2^ s^-1^ for HC, 3.0+/- 0.2 ×10^-3^ mm^2^ s^-1^ for FESZ) and RD values were increased (mean RD value 0.72+/-0.05 ×10^-3^ mm^2^ s^-1^ for HC, 0.78+/-0.07 ×10^-3^ mm^2^ s^-1^ for FESZ). Effect sizes for group differences in dMRI measures extracted from the AC (*d=-0.78* for FA; *d=0.89* for trace; *d= 0.94* for RD) were large (d≥0.8), according to Cohen's convention (Cohen, 1988).

### 3.4 4 Associations with medication

Most of the patients were treated with antipsychotic medication (mean chlorpromazine equivalent was 300 mg/day). To explore whether our findings were confounded by medication use, the chlorpromazine equivalents were correlated with the dMRI measures. Correlations of FA, trace and RD were small and not significant (Spearman rho for FA: *r*(13)= -0.28, *p* > 0.05, for trace: *r*(13)= 0.13, *p* > 0.05, and for RD: *r*(13)=0.19, *p* >0.05)

## 4. Discussion

In this study, we reconstructed the AC in FESZ and in healthy control subjects using dMRI (Fig.1) and found statistically significant differences between the two groups. More specifically, FA was reduced, trace and RD were increased and AD remained unchanged in FESZ (Fig. 2). AC has been explored in only one other schizophrenia study, which was also from our group (Choi et al., 2011). In that published study, AC was abnormal in patients diagnosed with chronic schizophrenia (Choi et al., 2011). In the current study, we report that abnormal AC is present early in the course of illness, where we document changes in FESZ patients primarily in FA and in RD. We interpret these observed changes in FA and in RD as indicating abnormal myelination of the AC in the early phases of schizophrenia. The findings from the chronic schizophrenia and our FESZ study are similar with respect to the changes in the dMRI measures and suggest aberrant myelination of the AC in chronic schizophrenia and in FESZ.

Abnormalities of axonal myelination are believed to be one of the features of schizophrenia that underpin disconnectivity of white matter in the brain. Abnormal myelination has been documented in post-mortem studies through altered oligodendrocytes, abnormalities in myelination of fibers, and changes in the expression profiles of myelin-related genes in the brains of patients diagnosed with schizophrenia (Hakak et al., 2001; Haroutunian and Davis, 2007; Haroutunian et al., 2007; Tkachev et al., 2003; Uranova et al., 2001). Studies using dMRI reported reduced FA and increased RD for several fiber tracts, including fornix, cingulum, inferior longitudinal fasciculus, anterior limb of the internal capsule, frontostriatal connections and corpus callosum, and reconfirmed abnormal myelination of axons in vivo (Abdul-Rahman et al., 2011; Ashtari et al., 2007; Carletti et al., 2012; Holleran et al., 2014; Levitt et al., 2012; Quan et al., 2013; Seal et al., 2008; Whitford et al., 2010). Our study, which reports decreases in FA and increases in trace and RD in the AC, extends the list of tracts impacted by abnormal myelination of the axons. Finally, myelin degeneration might be a secondary response to neuroinflammation, which has been hypothesized to occur in the phase of first clinical episode of psychosis (Pasternak et al., 2012).

This study has several limitations. First, all but two FESZ patients were taking antipsychotic medication either at the time of the scan or in the past, thus, as with previous studies, medication may confound the effects. Correlations of medication dosage with dMRI measures, however, were small and not statistically significant, suggesting that medications do not account for the findings reported here. Second, the AC tract was reconstructed in 16 out of 20 controls. The reason for loss of some controls (3 males, 1 female) was likely due to the small diameter of the AC. That is, the AC diameter is smaller than the voxel size (1.7 mm) afforded by our dMRI data. Third, the sample used was constituted mainly of right-handed subjects (100 % in healthy control group and 82% in FESZ group). Although handedness might possibly effect the distribution of homotropic and heterotropic connections within the AC, here we have not tested any hypothesis in respect to handedness. This would have to be investigated in studies using larger samples. Lastly, the number of subjects was relatively small, but the effect sizes were large, indicating the robustness of the findings.

In summary, we reconstructed the AC, an interhemispheric white matter fiber tract, from dMRI data. When compared to healthy controls we found structural abnormalities in the AC in FESZ patients. Based on the changes observed in dMRI indices of the AC, we interpret these novel findings in FESZ as indicative of abnormal myelination in the white matter, abnormalities found previously in the chronic stages of schizophrenia. Abnormalities of myelin in the AC likely interfere with its function as an interhemispheric pathway. Previous literature has demonstrated the importance of the compensatory role of the AC for interhemispheric communication and cognitive functions when connectivity through the CC is affected. Abnormalities in the CC have been reported previously in chronic and in FESZ patients. Concomitant abnormalities in the CC and the AC might well lead to reduced interhemispheric connectivity and deficits in cognition and should be addressed in future studies.

## Figures and Tables

**Table 1:** Demographic and clinical characteristics of study groups

		N	Mean	SD	p
**Age (years)**	FESZ	17	21.76	4.42	0.20
	HC	16	23.63	3.99	
**Gender (male;female)**	FESZ	17	13M;4F	N.A.	0.93
	HC	16	12M;4F	N.A.	
**Education (school years)**	FESZ	17	13.94	2.49	0.90
	HC	16	13.84	1.58	
**Socioeconomic status parent**	FESZ	17	2.53	1.55	0.18
	HC	16	1.94	0.77	
**Handedness (% right handed)**	FESZ	17	82%	N.A.	N.A.
	HC	16	100%	N.A.	
**WRAT-4 Reading subscore**	FESZ	17	116	15	0.8
	HC	6	114	17	
**Age of illness onset (years)**	FESZ	15	20.87	4.16	N.A.
**Duration of illness (years)**	FESZ	15	0.67	0.62	N.A.
Antipsychotic medication* (chlorpromazine aequivalenlt)	FESZ	15**	300	304	N.A.
**SAPS (total score)**	FESZ	17	18.41	18.14	N.A.
**SANS (total score)**	FESZ	16	28.41	14.91	N.A.
